# Testicular Leydig Cell Tumor with Metachronous Lesions: Outcomes after Metastasis Resection and Cryoablation

**DOI:** 10.1155/2015/748495

**Published:** 2015-10-07

**Authors:** Julio J. Geminiani, Stephen D. Marshall, Tammy S. Ho, Steven B. Brandes

**Affiliations:** Division of Urologic Surgery, Department of Surgery, School of Medicine, Washington University in St. Louis, 4960 Children's Place, Box 8242, Saint Louis, MO 63110, USA

## Abstract

Leydig cell tumors represent 3% of testicular masses and usually occur in prepubertal boys and men between 30 and 60 years of age. Leydig cell tumors are benign in children but can be malignant in 10% of adults. This case report describes a 41-year-old patient who was diagnosed with a Leydig cell tumor that originated in his right testicle that subsequently metastasized to his liver, lungs, and retroperitoneum. We discuss the patient's presentation and review the radiographic findings, surgical treatment, surgical pathology, chemotherapeutic treatment, and published literature on this rare pathology.

## 1. Introduction

The interstitial cells of Leydig were named after the German anatomist Franz von Leydig who first described them. They develop from the mesenchyme and primarily produce testosterone in response to luteinizing hormone (LH) as well as estrogens. Leydig cell tumors (LCTs) comprise only 1.2–3% of testicular neoplasms; however, up to 10% of LCTs are malignant. Malignant LCTs tend to progress at a rapid pace with median survival of 2 years after orchiectomy [1]. Management of patients after orchiectomy is controversial, as no standard of care exists. We present a case report of a patient who developed metastatic LCT and was managed initially with observation and subsequently with metastasectomies and cryoablation.

## 2. Case Presentation

**Figure 1:**
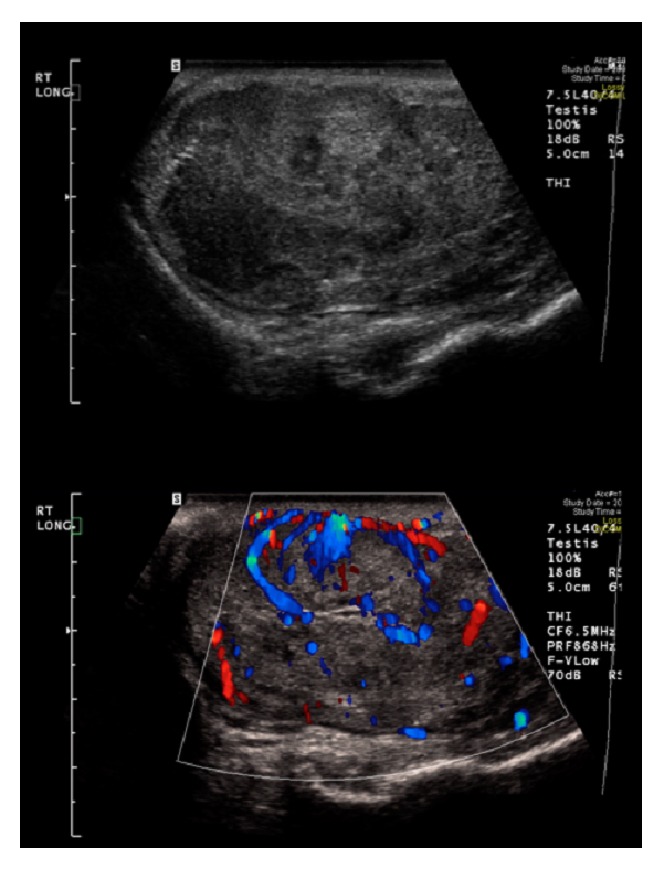
Right testicular ultrasound, showing a testicular mass with flow on Doppler.

**Figure 2:**
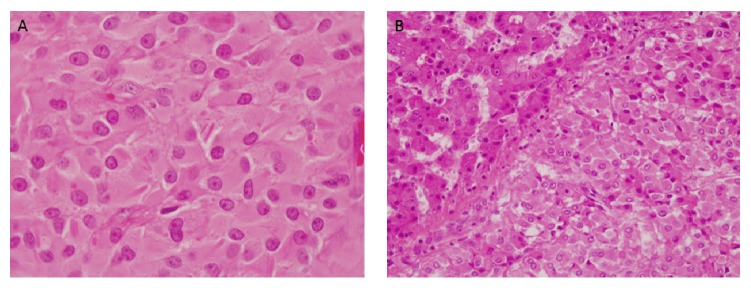
(A) High magnification photomicrograph of the testicular mass showing sheets of polygonal cells with abundant eosinophilic cytoplasm, distinct cell borders, and bland uniform nuclei with prominent nucleoli. Numerous Reinke crystals are present. (B) Photomicrograph of metastatic Leydig cell tumor (lower right) within the liver (upper left).

A 41-year-old male presented with a right-sided testicular mass. Physical examination revealed a firm, painless right-sided testicular mass with no gynecomastia. Testicular ultrasound showed a 6.2 × 3.6 × 4.4 cm intratesticular lesion suspicious for malignancy (Figure 1). Preoperative workup including tumor markers (*β*-human chorionic gonadotropin, alpha fetal protein, and lactate dehydrogenase), chest X-ray (CXR), and Abdomen and pelvis CT was normal. He underwent a right radical inguinal orchiectomy with pathology showing a 5.0 cm Leydig cell tumor with negative surgical margins (Figure 2(A)). Of note, there was moderate mitotic activity and tumor necrosis, though no lymphovascular invasion (LVI) or cellular atypia was noted. During subsequent follow-up, he began to experience fatigue, hot-flashes, and night sweats and was noted to have low testosterone at 180 ng/dL. FSH was 5.1 IU/L (1.4–18) and LH was 6 IU/L (2–9). His hypogonadism was attributed to suppression of his hypothalamic axis due to a testosterone secreting LCT. When his testosterone level did not improve to normal levels postoperatively, he was started on testosterone replacement therapy (200 mg IM every three weeks) which drastically improved his symptoms.

**Figure 3:**
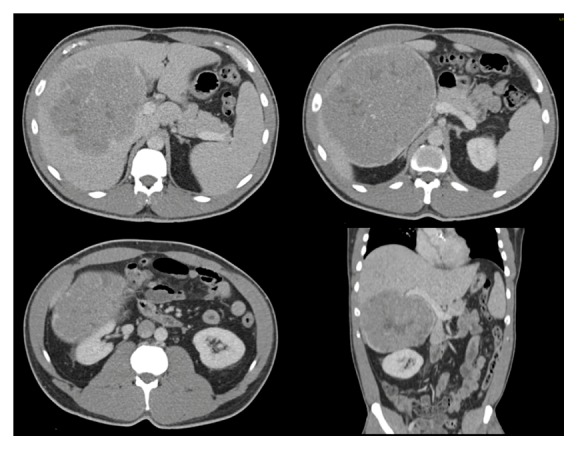
Abdomen and pelvis CT that shows a 16 cm mass in the right lobe of the liver, with mass effect on the surrounding tissues, with compression of the IVC.

Given the risk of malignancy, he was followed up with CXR and serum testosterone levels every six months for a year and then yearly. Three years after orchiectomy, the patient presented with right upper quadrant abdominal pain and was found to have a large 15 × 15 cm right hepatic lesion with mass effect on adjacent structures, including narrowing of the IVC on CT (Figure 3). Since his testosterone was 1600 ng/dL at the time, he was presumed to have metastatic LCT and subsequently an exploratory-laparotomy and right hepatectomy with a hepaticojejunostomy with Roux-en-Y reconstruction. Pathology confirmed metastatic LCT with negative surgical margins (Figure 2(B)). After the surgery, his testosterone level dropped to 205 ng/dL. Screening CT scans every 3 months for a year remained negative for metastasis so the patient resumed his testosterone replacement therapy in order to return to his baseline energy level and quality of life.

**Figure 4:**
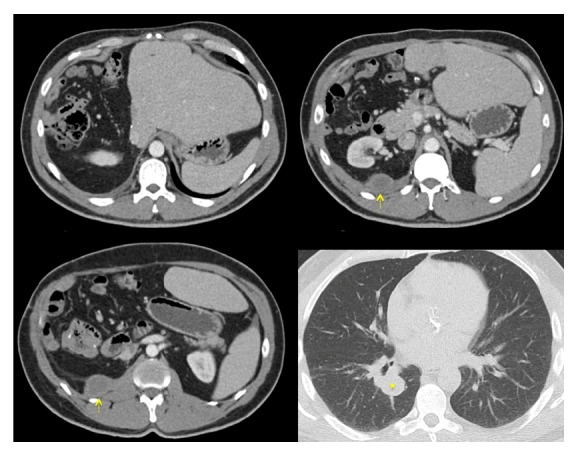
Abdomen and pelvis CT that shows a new retrocrural mass (arrows) and lower right pulmonary nodule (star). A previous right hepatectomy can be observed.

**Figure 5:**
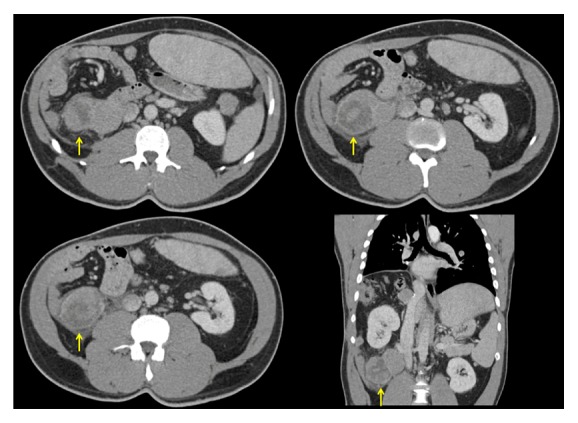
Abdomen and pelvis CT that shows a right-sided retroperitoneal recurrent mass prior to cryoablation.

**Figure 6:**
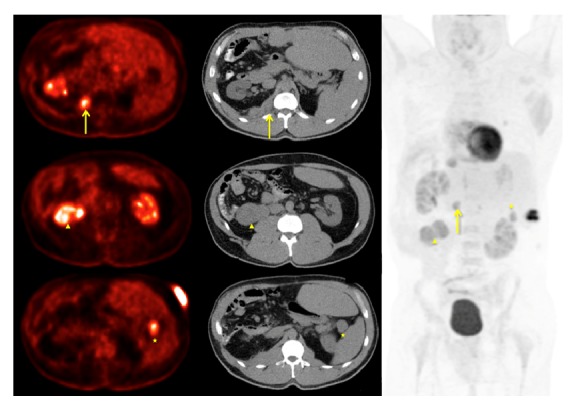
PET scan that demonstrates FDG avidity of omental lymph nodes (stars) and retrocrural lesions (arrows and arrows head).

Five years after his initial orchiectomy, his testosterone level abruptly increased to 1287 ng/dL. A CT scan showed a new right retrocrural mass and pulmonary node (Figure 4). The retrocrural mass was resected and he later underwent a video-assisted thoracoscopic surgery (VATS) of his right lower lobe pulmonary nodule. Pathology for both procedures redemonstrated metastatic LCT. A year later a follow-up CT scan revealed a new right-sided retroperitoneal mass which was treated with cryoablation (Figure 5). He remained disease-free for a year. Seven years after orchiectomy, CT/PET imaging noted FDG avid lesions on omental lymph nodes and left sided pulmonary nodules as well as liver lesions (Figure 6) with testosterone level of 6034 ng/dL. He then underwent four cycles of cisplatin and etoposide chemotherapy. Unfortunately, he experienced progression of disease with increasing testosterone to 8459 ng/dL and CT showing increasing disease burden.

## 3. Discussion

Leydig cell tumors (LCTs) represent 1–3% of testicular masses and can occur at any age but are found most commonly in prepubertal boys and men between the ages of 30 and 60 [2]. Tumor markers (hCG, AFP, and LHD) are typically negative, but endocrine anomalies such as elevated testosterone or estrogen are sometimes observed [1]. In a review of 32 patients with metastatic LCT, 54% of patients had elevated androgen levels and 50% of patients had elevated estrogen level. According to EUA Guidelines, standard tumor markers as well as LH, FSH, and testosterone levels should be obtained if there is clinical suspicion of LCT. If inconclusive, estrogen, estradiol, progesterone, and cortisol levels may be helpful. Elevated estrogen levels, which are a result of either direct production or indirect peripheral aromatization, can often present as gynecomastia in 10% of patients [3]. In our patient, we observed high serum testosterone levels with recurrence and used it as a surrogate during follow-up in addition to CXRs and cross-sectional imaging.

While LCTs are benign in children, they can be malignant in 10% of adults. Of those with malignant disease, 22% present with metastasis, whereas 19% of metastases develop within 12 months and 59% develop thereafter. In one review of 32 patients with LCTs, metastases most frequently involved the lymph nodes (72%), lung (43%), bone (28%), and kidney (14%) [1]. Aside from the presence of metastatic disease at diagnosis, there are few accurate predictors of aggressive disease. In a pathologic comparison between 25 benign and 5 malignant LCTs, Kim et al. found that patients with malignant tumors tended to have larger tumors greater than 5 cm, moderate or severe nuclear atypia, angiolymphatic invasion, infiltrating margins, or greater than 5 mitotic features per 10 high power fields [4].

Despite a known 10% malignancy rate, there are no standard protocols for either postorchiectomy surveillance or more aggressive treatment such as retroperitoneal lymph node dissection (RPLND). In a recent retrospective review of 48 patients with testicular sex cord-stromal tumors including 28 patients with LCTs, Silberstein et al. found that patients with 1 or no high risk pathologic features can be safely observed without RPLND [5]. Conversely, it appears that some patients with 2 or more risk factors with either clinical stage I or clinical stage IIa disease at diagnosis seemed to benefit from early RPLND. However, a subset of these patients developed relapse regardless of early versus delayed RPLND, indicating the progressive nature of LCTs. Given the limited utility of chemotherapy and radiation for metastatic LCTs [6], it appears that early RPLND may offer the possibility of a good outcome in the setting of low nodal burden.

Though there is no standard of therapy for management of metastatic LCTs, our limited experience has shown that surgical resection and minimally invasive approaches such as cryoablation may play an important role in treatment. To our knowledge, no case reports have managed retroperitoneal recurrence with cryoablation. Despite having several pathologic risk factors at diagnosis, including larger size, tumor necrosis, and moderate mitotic activity, our patient responded well initially to metastasectomies and cryoablation of retroperitoneal recurrence. He subsequently survived over seven years after orchiectomy before developing widely progressive disease in comparison to the typical median survival of two years after orchiectomy [1]. Additionally, cryoablation offers a less invasive means of management and avoids the morbidity associated with surgical resection, especially in patients who have already undergone extensive surgical resection. In particular, our patient had a previous Roux-en-Y and was able to quickly resume daily activities with minimal recovery time. However, this remains a rare tumor and more studies are needed to evaluate the efficacy of early RPLND versus delayed metastasectomy for the management of metastatic LCT.
